# 518. Model-informed Dose Selection of Dual Toll-like Receptor 7/8 Inhibitor Enpatoran (M5049) for the Treatment of COVID-19 Pneumonia

**DOI:** 10.1093/ofid/ofab466.717

**Published:** 2021-12-04

**Authors:** Lena Klopp-Schulze, Jamie Shaw, Jennifer Dong, Akash Khandelwal, Elizabeth Adams, Dongzi Yu, Kosalaram Goteti

**Affiliations:** 1 The healthcare business of Merck KGaA, Darmstadt, Germany, Darmstadt, Hessen, Germany; 2 EMD Serono, Billerica, MA, USA, Billerica, Massachusetts; 3 The healthcare business of Merck KGaA, Darmstadt, Hessen, Germany; 4 EMD Serono, Billerica, MA, USA; Current affiliation: BioNTech SE, Germany, Billerica, Massachusetts

## Abstract

**Background:**

Enpatoran, formerly known as M5049, is a potential first-in-class small molecule antagonist of toll-like receptors (TLR) 7 and 8, which may prevent viral-associated hyperinflammatory response and progression to ‘cytokine storm’ in coronavirus disease 2019 (COVID-19) patients. The objective of this study was to leverage existing population pharmacokinetic/pharmacodynamic (popPK/PD) models for enpatoran to inform dose selection for an accelerated Phase II study in COVID-19 patients with pneumonia.

**Methods:**

The popPK/PD models were based on plasma PK and PD biomarker (ex vivo-stimulated interleukin [IL]6 and interferon α [IFNα] secretion) data from the enpatoran first-in-human Phase I study in healthy participants (Port A, et al. Lupus Sci Med 2020;7(Suppl. 1): Abstract P135). A two-compartment model describing PK used a sigmoidal E_max_ model with proportional decrease from baseline characterizing the PD response across the investigated single and multiple daily dose range of 1–200 mg (N=72). Concentrations that inhibited 50% and 90% (IC_50_/IC_90_) of cytokine secretion were estimated and stochastic simulations were performed to assess target coverage under different dosing regimens.

**Results:**

Simulations suggested that, to achieve maximal inhibition of IL-6 over time, enpatoran PK concentrations would be maintained above the IC_90_ throughout the dosing interval with doses of 100 mg and 50 mg twice daily in 90% and 30% of participants, respectively. In comparison, IFNα inhibition was predicted to be lower, with IC_90_ coverage in 60% and 8% of participants with twice daily doses of 100 mg and 50 mg enpatoran, respectively.

**Conclusion:**

Utilization of existing popPK/PD models allowed for the accelerated development of enpatoran in COVID-19 to address an unprecedented global pandemic. Rational model-informed dose selection was supported by data from a Phase I study in which there were no safety concerns.

**Disclosures:**

**Lena Klopp-Schulze, PhD**, Merck KGaA, Darmstadt, Germany (Employee) **Jamie Shaw, BS**, EMD Serono Research & Development Institute, Inc., Billerica, MA, USA (an affiliate of Merck KGaA, Darmstadt, Germany) (Employee) **Jennifer Dong, PhD**, EMD Serono Research & Development Institute, Inc., Billerica, MA, USA (an affiliate of Merck KGaA, Darmstadt, Germany) (Employee) **Akash Khandelwal, PhD**, Merck KGaA, Darmstadt, Germany (Employee, Shareholder) **Elizabeth Adams, MD**, BioNTech SE, Germany (Employee)EMD Serono Research & Development Institute, Inc., Billerica, MA, USA (an affiliate of Merck KGaA, Darmstadt, Germany)(employer at the time of study) (Employee) **Dongzi Yu, MD**, EMD Serono Research & Development Institute, Inc., Billerica, MA, USA (an affiliate of Merck KGaA, Darmstadt, Germany) (Employee) **Kosalaram Goteti, PhD**, EMD Serono Research & Development Institute, Inc., Billerica, MA, USA (an affiliate of Merck KGaA, Darmstadt, Germany) (Employee)**Pfizer** (Shareholder)

